# Inflammatory Response to Burn Trauma: Nicotine Attenuates Proinflammatory Cytokine Levels

**Published:** 2014-12-19

**Authors:** L. Claassen, S. Papst, K. Reimers, C. Stukenborg-Colsman, L. Steinstraesser, P. M. Vogt, T. Kraft, A. D. Niederbichler

**Affiliations:** ^a^Department of Orthopedics, Hand and Reconstructive Surgery, Hannover Medical School, Hannover; ^b^Department of Anesthesiology, Hand and Reconstructive Surgery, Hannover Medical School, Hannover; ^c^Department of Plastic, Hand and Reconstructive Surgery, Hannover Medical School, Hannover; ^d^Department of Plastic, Reconstructive and Aesthetic Surgery, Handsurgery, European Medical School, Oldenburg; ^e^Department of Molecular and Cell Physiology, Hannover Medical School, Hannover; ^f^Department of Hand and Plastic Surgery, Helios Klinikum Berlin-Buch GmbH, Berlin, Germany

**Keywords:** burn trauma, nicotine, inflammatory reflex, neuroimmunology, cytokines

## Abstract

**Objective:** The immune response to an inflammatory stimulus is balanced and orchestrated by stimulatory and inhibitory factors. After a thermal trauma, this balance is disturbed and an excessive immune reaction with increased production and release of proinflammatory cytokines results. The nicotine-stimulated anti-inflammatory reflex offsets this. The goal of this study was to verify that transdermal administration of nicotine downregulates proinflammatory cytokine release after burn trauma. **Methods:** A 30% total body surface area full-thickness rat burn model was used in Sprague Dawley rats (n = 35, male). The experimental animals were divided into a control group, a burn trauma group, a burn trauma group with additional nicotine treatment, and a sham + nicotine group with 5 experimental animals per group. The last 2 groups received a transdermal nicotine administration of 1.75 mg. The concentrations of tumor necrosis factor alpha, interleukin 1 beta, and interleukin 6 were determined in homogenates of hearts, livers, and spleens 12 or 24 hours after burn trauma. **Results:** Experimental burn trauma resulted in a significant increase in cytokine levels in hearts, livers, and spleens. Nicotine treatment led to a decrease of the effect of the burn trauma with significantly lower concentrations of tumor necrosis factor alpha, interleukin 1 beta, and interleukin 6 compared to the trauma group. **Conclusions:** This study confirms in a standardized burn model that stimulation of the nicotinic acetylcholine receptor is involved in the regulation of effectory molecules of the immune response. Looking at the results of our study, further experiments designed to explore and evaluate the potency and mechanisms of the immunomodulating effects of this receptor system are warranted.

Severe burn trauma induces a proinflammatory immune response. The increased production and release of proinflammatory cytokines like tumor necrosis factor alpha (TNF-α), interleukin 1 beta (IL-1β), and interleukin 6 (IL-6) is an eminent pathogenic factor.^1^^,^^2^ The concentrations of these proinflammatory cytokines reach their maximum levels between 12 and 24 hours after a burn trauma.^1^

There is a communication between the nervous and the immune system.^3^^,^^4^ The central nervous system has a crucial influence on immune responses via the vagal nerve.^5^^,^^6^ Tracey termed the complex immune response to parasympathetic stimuli the “inflammatory reflex.”^7^ The efferent part of this reflex was named the “cholinergic anti-inflammatory pathway” because of its identified primary neurotransmitter acetylcholine.^8^ The vagus nerve innervates most of the internal organs, including those that contain parts of the reticuloendothelial system such as the liver, spleen, and heart. The target cells for the anti-inflammatory effect are primarily macrophages, which are effectively inhibited.^5^^,^^6^

The inflammatory reflex can be stimulated pharmacologically by acetylcholine and in addition by the receptor agonist nicotine.^9^ Besides the “classical” alveolar resorption of nicotine (inhalation of tobacco smoke), the transdermal absorption is described. The transdermal application of nicotine was developed in the context of smoking cessation and was used experimentally in studies of several research groups.^10^^-^12 Anti-inflammatory effects of nicotine are supposed to be mediated via the nicotinic acetylcholine receptor (nAChR) of the parasympathetic nerve.^13^ The nAChR was found in many organ systems and cells. Macrophages and monocytes express nAChR in their cell membrane. Activation leads to decreased endocytic and phagocytic activity. This results in a reduced antigen presentation, which is important for the initialization of an immune response.^13^^,^^14^

The present study was conducted to evaluate the potential effects of transdermal nicotine treatment on the inflammatory response after burn trauma.

## MATERIAL AND METHODS

### Study design

The study was performed with male 300 g to 350 g Sprague Dawley rats from Charles River. The experiments were done according to the standards and provisions of the Animal Welfare Act and the German Society for Experimental tests on animals (study protocol no. 05/1052). The university committee for the use and protection of animals and the Lower Saxony State Office for Consumer Protection and Food Safety (LAVES, Oldenburg, Germany) approved the protocol of the experiments.

**Figure 1:**
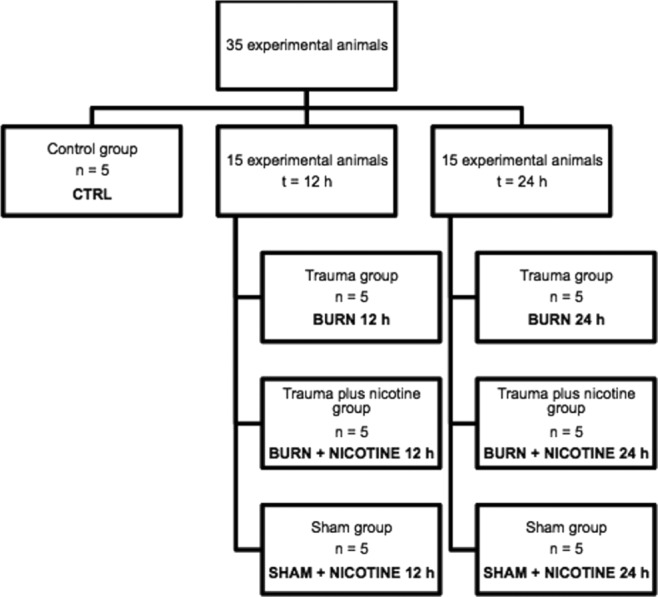
Experimental groups. The classification of 35 experimental animals into 7 experimental groups with 5 experimental animals each is depicted. We compared cytokine levels 12 hours and 24 hours after experimental burn trauma to a control group.

Seven study groups, each of n = 5 animals, were created. One control group was carried in the study (CTRL). The other 6 groups were divided into 3 groups in which the removal of organs were performed after 12 hours, and 3 groups in which this was done after 24 hours. At these 2 time points, a burn trauma group (BURN 12 hours and BURN 24 hours), a trauma group plus nicotine administration via nicotine patch (BURN + NICOTINE 12 hours and BURN + NICOTINE 24 hours) and a sham control group with nicotine application (SHAM + NICOTINE 12 hours and SHAM + NICOTINE 24 hours) were added (Fig 1).

### Burn trauma procedure and fluid resuscitation

Under isoflurane anesthesia dorsal hair was closely clipped. The rats were then placed in a prefabricated mold device with a rectangular opening of 2.1 cm^2^ that exposed the dorsal skin surface while protecting the remaining skin from burn exposure. The exposed skin surface was immersed in 60°C water for 40 seconds producing a full-thickness dermal burn of 30% total body surface area as previously described.^15^ Sham animals underwent an identical procedure, except that they were immersed in room temperature water (24°C). Control animals received no room temperature water immersion.

### Nicotine application

Nicotine was applied using a nicotine patch system (Nicorette, Fa. Pfizer Consumer Healthcare, Karlsruhe, Germany). The size of the nicotine patch was chosen on the basis of a study of Kalra and colleagues using cotinine serum levels reflecting strong smoking habits, which has been shown to range around 850 ng/mL.^16^ The patch released a nicotine dose of 1.75 mg. Animals of BURN + NICOTINE and SHAM + NICOTINE groups received nicotine treatment.

### Tissue isolation and cytokine secretion analysis (Enzyme-linked immunosorbent assay)

Under isoflurane anesthesia, a laparotomy was performed using a midline incision. Subsequently, a bilateral thoracotomy flap was created and the heart was rapidly excised and rinsed in ice-cold, isotonic, and sterile phosphate-buffered saline. Tissue pieces of the left ventricle, liver, and spleen were excised, weighed, and immediately frozen in liquid nitrogen for later processing.

Whole blood was allowed to clot for 2 hours at room temperature and was then spun down at 1000 g and 4°C for 20 minutes to separate serum from corpuscular elements, and the serum supernatant was pipetted off and frozen away for later assessment.

Hundred milligram of tissue was homogenized in 1000 μL ice-cold RIPA buffer consisting of 0.3 M NaCl, 20 mM Tris-HCl and pH 8.1% Sodium deoxycholate, 0.1% SDS, 1% Triton X-100, 1 mM EDTA, 1 mM PMSF, and protease inhibitor (Complete X, Roche, Indianapolis, Indiana). Homogenates were centrifuged at 3000 g for 5 minutes at 48°C and supernatant collected and stored at −80°C until use. Rat TNF-α, IL-1β, and IL-6 were measured by sandwich enzyme-linked immunosorbent assay (ELISA) using commercially available antibodies and standards as specified in the manufacturer's instructions (Quantikine, R&D Systems, Wiesbaden, Germany). Cotinine, the degradation product of nicotine, was determined in serum samples (Fa. Calbiotech, Spring Valley, California). Optical density was assessed using an automated plate reader set at a wavelength of 450 nm with a correction reading of 540 nm (Infinite 200, TECAN GmbH, Crailsheim, Germany). Cytokine concentrations were determined from the standard curve and expressed as pg/mL.

### Statistical analysis

Statistical analysis was done using Prism 5 software (GraphPad Inc, La Jolla, California). Analysis of variance (ANOVA) followed by Tukey's post hoc test was used. Statistical significance was set at *P* ≤ .05. The results were expressed as means ± standard deviation (SD). To enhance the clearness of our figures, we only highlighted the results of the statistical analysis between CTRL and BURN group and between BURN and BURN + NICOTINE group. The statistical analysis was performed in accordance with our institute for biometry.

## RESULTS

### Serum cotinine concentration

**Figure 2:**
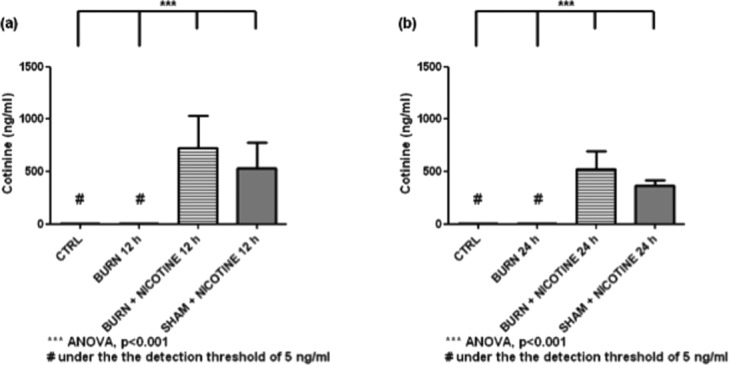
Serum cotinine concentration. Increase of cotinine concentrations in sera of experimental animals with transdermal nicotine application (striped and gray bars). In contrast, cotinine concentrations remained below the detection limit in the sera of experimental animals without nicotine application. Cotinine values are expressed as mean + SD. Each bar represents n = 5 experiments.

The cotinine concentrations of the CTRL and BURN groups were below the detection limit of the ELISA of 5 ng/mL. In contrast, the values of the experimental animal groups with nicotine application were increased significantly (Figs 2a and 2b, white and black bars vs striped and gray bars, *P* < .001). After 24 hours, the values of the respective groups were lower than those after 12 hours, the differences were not statistically significant.

### Cytokine concentrations in organ homogenates

**Figure 3:**
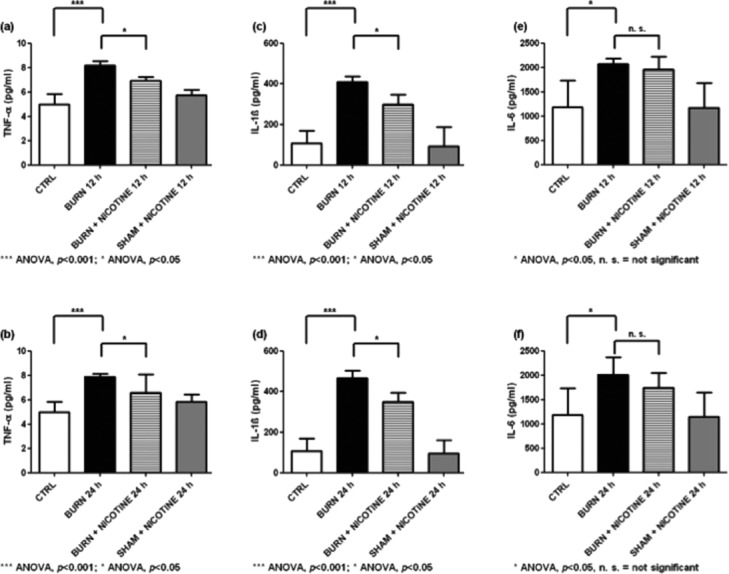
Cytokine concentrations of heart homogenates: (*a*) and (*b*) TNF-α concentrations in heart homogenates. (*c*) and (*d*) IL-1β levels in heart homogenates. (*e*) and (*f*) IL-6 concentrations in heart homogenates. In each diagram, the significant increase of cytokines due to the experimental burn injury compared to the control group is marked (black bars vs white bars). Also the reduction of the increased cytokine levels through transdermal nicotine application was significant for TNF-α and IL-1β (black columns vs striped columns). The values are expressed as mean + SD. Each bar represents n = 5 experiments.

Heart homogenate analysis of TNF-α showed a marked rise of TNF-α levels in BURN compared to CTRL and SHAM + NICOTINE animals. The BURN 12-hour group had 8.6 ± 0.1 pg/mL and the BURN 24-hour group had 7.9 ± 0.1 pg/mL (Figs 3a and 3b, black bars, ANOVA, *P* < .001). Nicotine application induced a significant decrease in heart TNF-α production (Figs 3a and 3b, black vs striped bars, ANOVA, *P* < .05). The TNF-α concentration for the BURN + NICOTINE 12-hour group was 6.9 ± 0.1 pg/mL and for the BURN + NICOTINE 24-hour group was 6.6 ± 0.7 pg/mL.

In heart homogenates that were assayed for IL-1β, we found a significant increase in tissue derived from BURN groups compared to their SHAM + NICOTINE and CTRL counterparts (Figs 3c and 3d, black bars vs gray and white bars, ANOVA, *P* < .001). Burn injury followed by nicotine application induced a significantly lower heart IL-1β production compared to nontreated burn trauma animals; however, the IL-1β levels of BURN + NICOTINE were still significantly higher than for CTRL animals (Figs 3c and 3d, striped vs white bars, ANOVA, *P* < 0.05).

Interleukin 6 concentrations reached concentrations of around 2200 pg/mL in heart homogenates of burn-injured animals (Figs 3e and 3f, black bars). The average baseline IL-6 level in control animals was found to be approximately 1200 pg/mL (Figs 3e and 3f, white bars). Nicotine application induced a slight reduction of produced IL-6 compared to BURN animals.

**Figure 4:**
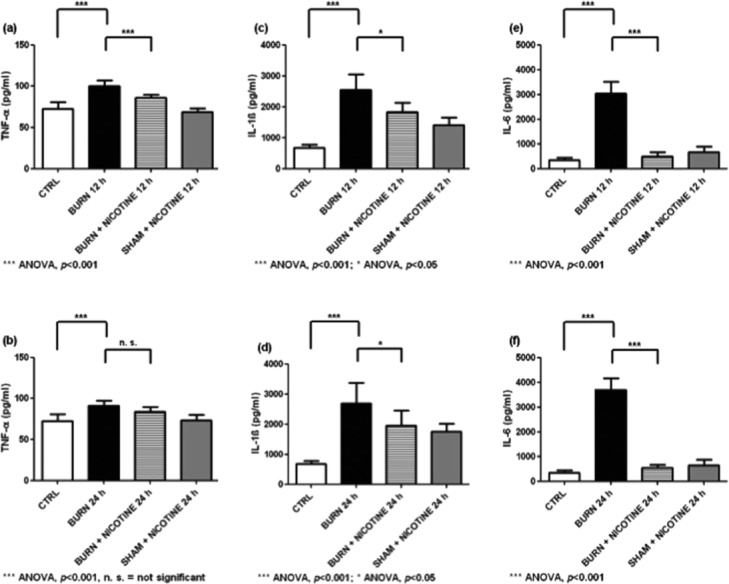
Cytokine concentrations of liver homogenates: (*a*) and (*b*) TNF-α concentrations in liver homogenates. (*c*) and (*d*) IL-1β levels in liver homogenates. (*e*) and (*f*) IL-6 concentrations in liver homogenates. The experimental burn injury led to significantly increased cytokine levels compared to the control group (black vs white bars). Also the reduction of the burn trauma induced increase of the cytokine concentrations due to transdermal nicotine application was statistically relevant except for TNF-α after 24 hours (striped vs black bars). The values are expressed as mean + SD. Each bar represents n = 5 experiments.

All cytokine experiments in liver homogenates showed a significant increase of the evaluated cytokine when animals that underwent burn trauma were compared with SHAM + NICOTINE or CTRL animals. In liver homogenates, we found baseline TNF-α concentrations around 70 pg/mL in control animals and similar values also in SHAM + NICOTINE animals (Figs 4a and 4b, gray and white bars). Liver homogenates from burn-injured animals revealed mean TNF-α concentrations of 104.4 ± 2.3 pg/mL after 12 hours and 91.4 ± 2.0 pg/mL after 24 hours (Figs 4a and 4b, black bars, ANOVA, *P* < .001). In contrast, the BURN + NICOTINE groups had decreased values with 86.1 ± 2.3 pg/mL and 83.6 ± 2.4 pg/mL (Figs 4a and 4b, striped bars, after 12 hours ANOVA, *P* < .001, not significant after 24 hours).

A significant reduction of IL-1β levels was observed when we compared liver homogenate ELISA results from burn alone with BURN + NICOTINE animals (Figs 4c and 4d, black and striped bars, ANOVA, *P* < .05). Interleukin 1 beta levels of BURN and BURN + NICOTINE groups did not reach baseline CTRL levels (Figs 4c and 4d, black and striped bars vs white bar).

Looking at IL-6 concentrations in liver homogenates after burn trauma, we found mean values of 3000 to 4000 pg/mL (Figs 4e and 4f, black bars). When burn-injured animals underwent nicotine application, IL-6 concentrations in liver homogenates were significantly decreased compared to burn-only animals (Figs 4e and 4f, striped vs black bars, ANOVA, *P* < 0.001).

**Figure 5:**
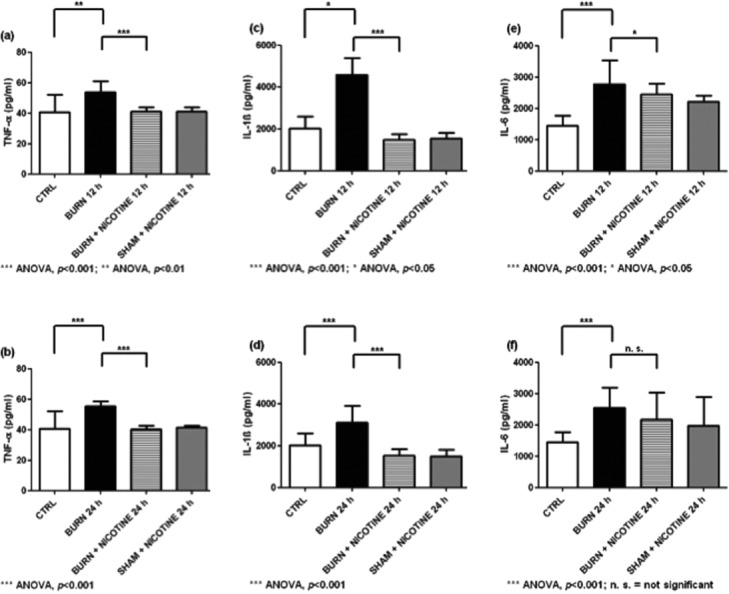
Cytokine concentrations of spleen homogenates: (*a*) and (*b*) TNF-α levels in spleen homogenates. (*c*) and (*d*) IL-1β levels in spleen homogenates. (*e*) and (*f*) IL-6 levels in spleen homogenates. Likewise to heart and liver homogenates burn trauma results in significantly increased levels of proinflammatory cytokines in spleen homogenates (black vs white bars). Also the reduction in the burn trauma induced increase of the cytokine concentrations due to transdermal nicotine application was significant except for IL-6 after 24 hours (striped vs black bars). The values are expressed as mean + SD. Each bar represents n = 5 experiments.

The highest TNF-α levels in spleen homogenates were found in the BURN groups, with values of 53.7 ± 2.3 pg/mL for the BURN 12-hour group and 55.5 ± 1.0 pg/mL for the BURN 24-hour group (Figs 5a and 5b, black bars). The experimental burn injury led to significantly increased IL-1β and IL-6 levels in the spleen homogenates. In all cases except for IL-6 after 24 hours, the transdermal nicotine application resulted in significantly decreased cytokine concentrations (Figs 5a to 5f, striped vs black bars).

## DISCUSSION

Severe thermal injury induces multiple pathophysiological changes leading to death and disability in severely burned patients. The multifactorial pathogenesis of burn-induced multiple organ dysfunction has extensively been described in the literature. A crucial pathogenic factor is – globally – an imbalance of the immune system.^1^ With the present study, we itemize the information about release of TNF-α, IL-1β, and IL-6 after burn trauma to 3 relevant organs. Furthermore, we can show that nicotine application is sufficient to attenuate the concentrations of these proinflammatory cytokines after burn trauma.

Our own previous results and the results of others have demonstrated that electric parasympathetic stimulation, for example, vagus nerve stimulation, also induces attenuation of inflammatory response markers. The direct electrical stimulation of the vagal nerve achieved an anti-inflammatory effect that is in turn reflected by the decreased levels of TNF-α, IL-1β, and IL-6 in the BURN + NICOTINE subsets.^9^^,^^17^^,^^18^ In the present study, the inflammatory reflex was pharmacologically induced using transdermal nicotine application with a commercially available patch. The pharmacological stimulation by peroral and transdermal nicotine administration has demonstrated its anti-inflammatory action. Clinically, this has been demonstrated in patients with chronic inflammatory bowel disease.^10^^,^^11^ The pharmacological stimulation via transdermal nicotine application has additional advantages. Clinical experience with this treatment approach exists and it is not invasive.

Nevertheless, the isolated nicotine application cannot be compared with nicotine application by smoking tobacco products. Tobacco smoke contains more than 4800 toxic and carcinogenic substances. Ninety of these ingredients have been identified as carcinogens.^19^ It must be emphasized that the results shown in the literature as well as the results of this study are generated by the sole administration of nicotine and therefore must be clearly discerned from the harmful effects of tobacco smoke.

TNF-α, IL-1β, and IL-6 have been described as proinflammatory cytokines. Their suitability as indicators of the activity or the effect of the immune response after a burn trauma has been demonstrated by numerous previous studies.^18^^,^^20^^-^22 Despite extensive research on the kinetics of the inflammatory response after a burn trauma, the concentrations of these cytokines in organs such as heart, liver, and spleen after burn trauma remained unknown.

In homogenates of heart, liver, and spleen, significantly increased TNF-α, IL-1β, and IL-6 levels were found. These results correlate with data published previously by Gauglitz and colleagues.^1^ This group detected increased IL-1β and IL-6 concentrations in serum after 12 hours and 24 hours after burn injury. In homogenates of isolated cardiomyocytes, increased levels of TNF-α, IL-1β, and IL-6 were found at different time points, including 12 hours and 24 hours after burn injury.^2^ Furthermore, in isolated cardiomyocytes of rats, the TNF-α, IL-1β, and IL-6 concentrations were increased rapidly after infliction of the trauma or the inflammatory challenge, respectively.^18^^,^^22^ In models using in vitro endotoxin challenge, increased cytokine levels were also reported in liver homogenates 3 hours after burn trauma.^18^ In this study, transdermal nicotine administration reduced the posttraumatic increased cytokine levels in the heart and liver homogenates (Figs 3a-f and 4a-f).

Our data provide evidence for positive effects of nicotine on posttraumatic hyperinflammation. Transdermal application of nicotine led to significantly increased levels of serum cotinine and reduced proinflammatory cytokine concentrations. However, the exact mode of action of nicotine is still unclear. It would be of great interest to clarify whether nicotine might reduce the lethality of patients with burn trauma. Thus, the results should prompt further studies on the topic of the inflammatory reflex and the treatment of burn patients.

## References

[1] Gauglitz GG, Song J, Herndon DN (2008). Characterization of the inflammatory response during acute and post-acute phases after severe burn. Shock.

[2] Maass DL, White J, Horton JW (2002). IL-1beta and IL-6 act synergistically with TNF-alpha to alter cardiac contractile function after burn trauma. Shock.

[3] Blalock JE (2005). The immune system as the sixth sense. J Intern Med.

[4] Andersson J (2005). The inflammatory reflex—introduction. J Intern Med.

[5] Czura CJ, Tracey KJ (2005). Autonomic neural regulation of immunity. J Intern Med.

[6] Tracey KJ (2007). Physiology and immunology of the cholinergic anti-inflammatory pathway. J Clin Invest.

[7] Tracey KJ (2002). The inflammatory reflex. Nature.

[8] Borovikova LV, Ivanova S, Zhang M (2000). Vagus nerve stimulation attenuates the systemic inflammatory response to endotoxin. Nature.

[9] Bernik TR, Friedman SG, Ochani M (2002). Pharmacological stimulation of the cholinergic antiinflammatory pathway. J Exp Med.

[10] Sandborn WJ (1999). Nicotine therapy for ulcerative colitis: a review of rationale, mechanisms, pharmacology, and clinical results. Am J Gastroenterol.

[11] Guslandi M (1999). Nicotine treatment for ulcerative colitis. Br J Clin Pharmacol.

[12] Richardson CE, Morgan JM, Jasani B (2003). Effect of smoking and transdermal nicotine on colonic nicotinic acetylcholine receptors in ulcerative colitis. QJM.

[13] de Jonge WJ, Ulloa L (2007). The alpha7 nicotinic acetylcholine receptor as a pharmacological target for inflammation. Br J Pharmacol.

[14] Wang H, Yu M, Ochani M (2003). Nicotinic acetylcholine receptor alpha7 subunit is an essential regulator of inflammation. Nature.

[15] Steinstraesser L, Fohn M, Klein RD (2001). Feasibility of biolistic gene therapy in burns. Shock.

[16] Kalra R, Singh SP, Pena-Philippides JC, Langley RJ, Razani-Boroujerdi S, Sopori ML (2004). Immunosuppressive and anti-inflammatory effects of nicotine administered by patch in an animal model. Clin Diagn Lab Immunol.

[17] Altavilla D, Guarini S, Bitto A (2006). Activation of the cholinergic anti-inflammatory pathway reduces NF-kappab activation, blunts TNF-alpha production, and protects against splanchic artery occlusion shock. Shock.

[18] Niederbichler AD, Papst S, Claassen L (2009). Burn-induced organ dysfunction: vagus nerve stimulation attenuates organ and serum cytokine levels. Burns.

[19] Nair U, Thielmann HW, Pötschke-Langer M (2009). Deutsches Krebsforschungszentrum (Hrsg.): Krebserzeugende Substanzen im Tabakrauch.

[20] Sambol JT, White J, Horton JW, Deitch EA (2002). Burn-induced impairment of cardiac contractile function is due to gut-derived factors transported in mesenteric lymph. Shock.

[21] Horton JW (2004). Left ventricular contractile dysfunction as a complication of thermal injury. Shock.

[22] Niederbichler AD, Westfall MV, Su GL (2006). Cardiomyocyte function after burn injury and lipopolysaccharide exposure: single-cell contraction analysis and cytokine secretion profile. Shock.

